# Real-time public health communication of local SARS-CoV-2 genomic epidemiology

**DOI:** 10.1371/journal.pbio.3000869

**Published:** 2020-08-21

**Authors:** Chaney C. Kalinich, Cole G. Jensen, Peter Neugebauer, Mary E. Petrone, Mario Peña-Hernández, Isabel M. Ott, Anne L. Wyllie, Tara Alpert, Chantal B. F. Vogels, Joseph R. Fauver, Nathan D. Grubaugh, Anderson F. Brito

**Affiliations:** 1 Department of Epidemiology of Microbial Diseases, Yale School of Public Health, New Haven, Connecticut, United States of America; 2 Department of Microbial Pathogenesis, Yale School of Medicine, New Haven, Connecticut, United States of America

## Abstract

Genomic epidemiology can provide a unique, real-time understanding of SARS-CoV-2 transmission patterns. Yet the potential for genomic analyses to guide local policy and community-based behavioral decisions is limited because they are often oriented towards specially trained scientists and conducted on a national or global scale. Here, we propose a new paradigm: Phylogenetic analyses performed on a local level (municipal, county, or state), with results communicated in a clear, timely, and actionable manner to strengthen public health responses. We believe that presenting results rapidly, and tailored to a non-expert audience, can serve as a template for effective public health response to COVID-19 and other emerging viral diseases.

## Abstract 2

This Perspective article proposes a new paradigm to present SARS-CoV-2 phylogenetic analyses performed on a local level (municipal, county, or state), with results communicated in a clear, timely, and actionable manner. The authors believe that presenting results rapidly, and tailored to a non-expert audience, can serve as a template for effective public health communication for COVID-19 and other emerging viral diseases.

## Genomic epidemiology

Pathogen genomics has played a key role in understanding the emergence and rapid global spread of SARS-CoV-2. Since the beginning of the COVID-19 pandemic, viral genomes have been generated at an unprecedented rate, with over 60,000 sequences on GISAID by early July 2020 (gisaid.org). Genomic epidemiology, using pathogen genomes to explore patterns of transmission, is a growing field. Genomic epidemiological analyses infer evolutionary relationships among pathogens using genome sequences and phylogenetic approaches [1]. These analyses can now occur in near real-time [1,2] and have yielded important insights into the early COVID-19 pandemic in the United States, Brazil, and South Africa [3–7]. In the context of a devolved public health system, genomic epidemiology of SARS-CoV-2 performed at a local level can provide (at least) two key insights. First, it can reveal how transmission transcends specific administrative boundaries. Second, it can capture idiosyncratic factors driving transmission, such as patterns of human movement and local and state policies, to quickly provide the type of actionable targets for mitigation strategies that generalized models or country-level analyses cannot achieve.

Hyper-local, situational sources of spread have been shown to play a crucial role in driving outbreaks of other respiratory illnesses [8]. Genomic epidemiology has demonstrated potential to elucidate these sources. Effective deployment of rapid sequencing and analysis for public health interventions depends on researchers’ ability to clearly and accurately convey results, their implications, and knowledge gaps within an actionable timeframe [9]. Yet the majority of these detailed genomic epidemiological analyses are finished weeks after the outbreaks, and are not updated as new data arrives. They are intended to be case studies aimed at understanding how viruses spread through an area, providing information with potential application to future outbreaks [1]. Such delay, however, hinders the use of genomic epidemiology to inform the response to viral outbreaks as they unfold.

Some fast-evolving RNA viruses can spread rapidly, but their genetic changes (mutations) do not always accumulate at the same speed as viral transmission. This means that when viral genomes are sequenced during short timeframes as outbreaks unfold, their sequences can be remarkably similar. This low genetic resolution often leads to uncertainties in the phylogenetic reconstructions, generating nuanced results that require careful analysis to prevent misinterpretations [10]. Some of these concerns can be mitigated by pairing phylogenetic findings with the best available epidemiologic data and other supporting data [10]. Despite these trade-offs between speed and accuracy, real-time phylogenetic analyses that are clearly communicated to the general public can support policies to prevent further spread, and foster actions and behavior changes based on scientific evidence.

## Science communication

A number of websites have played key roles in communicating the dynamics of SARS-CoV-2 spread in real-time by aggregating, analyzing, and displaying data. Dashboards with aggregated data such as case counts, tests, and hospitalizations became popular [11]. These dashboards often communicate data at global, national, and in the US, state and county levels, but do not (yet) incorporate any genomic data or analysis. Weekly situation reports of SARS-CoV-2 genomic epidemiology on global and continental levels have been published by Nextstrain (nextstrain.org) since January 23, 2020 [12], presenting the status of the pandemic across large geographical regions. Given the breadth of data covered by Nexstrain, however, there is a need for more fine-scale analysis and communication to impact policies at a local scale, as conducted by initiatives in the UK (COG-UK, cogconsortium.uk/news); Brazil (CADDE project, caddecentre.org/covid19) [6]; and South Africa (KRISP, krisp.org.za/covidnews.php) [7]. With these initiatives in mind, we propose three guiding principles for genomic epidemiologists looking to communicate their locally focused results in the context of the current COVID-19 pandemic (Box 1).

**Box 1. Guiding principles for public communication of genomic epidemiology finding**

1. **Communicate methods, analyses, and results in an accessible way.** For effective policy implementation, individual behavior adjustments, and to build trust in public health guidance, it is vital that genomic epidemiology findings are widely accessible, communicated in a jargon-free language, with appealing data visualizations and multilingual content (as appropriate).
2. **Speed must be balanced with accuracy, and uncertainty must also be communicated clearly.** Patterns revealed by available data may be inconclusive, or require more time or data to be informative. Even so, we believe that the benefit of sharing data when it can be acted upon in real-time outweighs the potential harm, as long as the uncertainties are explained. It also engages the public in critically thinking about the public health response to a rapidly developing crisis.
3. **Results should be communicated at a local level, with actionable conclusions.** Rapid insight is useful only insofar as it is also communicated rapidly and clearly to those with the power to act on it. Local public health implications explicitly tied to policy and behavior should be a consistent focus of any report.

## Case Study: CovidTrackerCT

**Fig 1:**
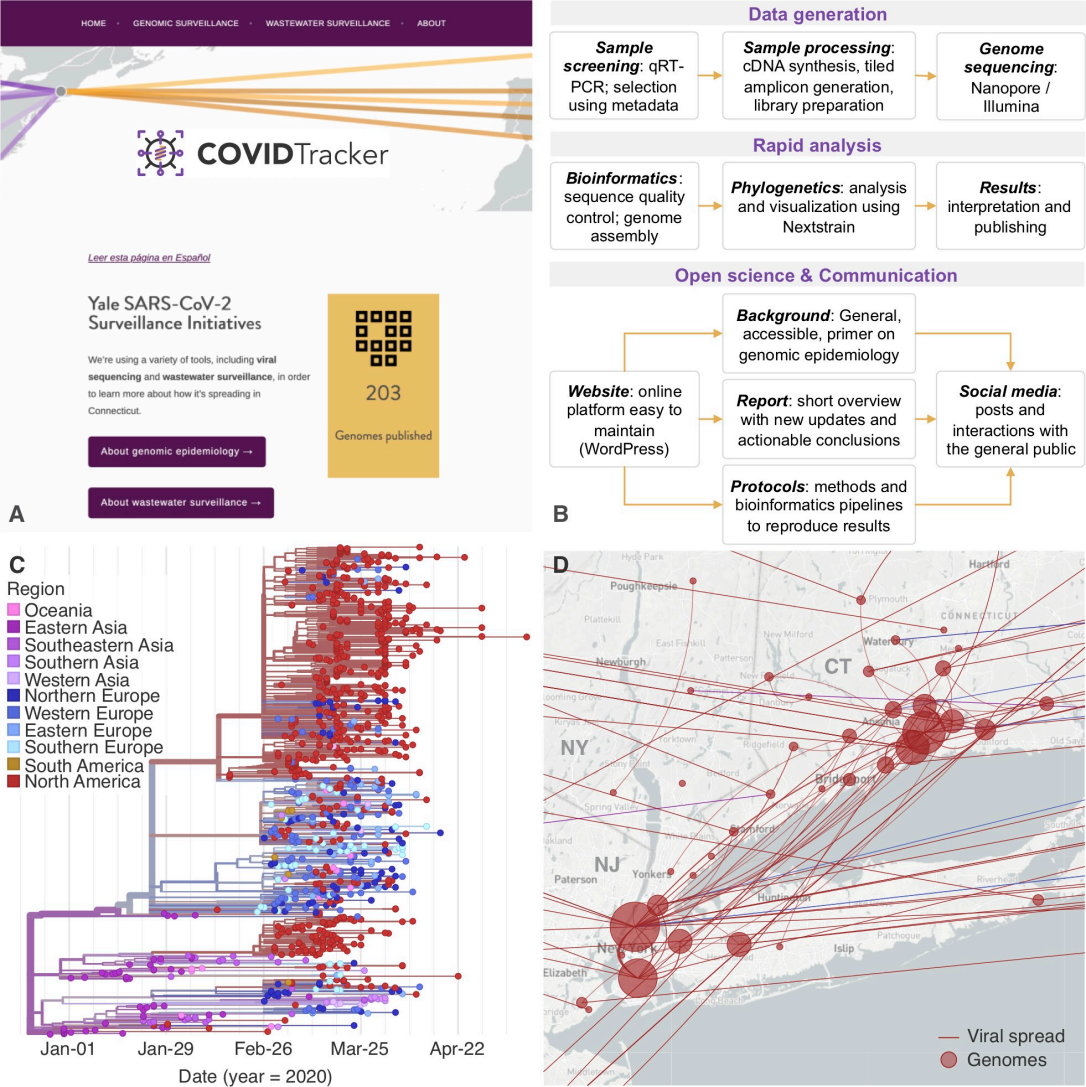
Open Science and Communication. (**A**) The COVIDTracker home page (coviditrackerct.com). Using an easy to maintain online platform, background information and regular reports are shared with the general public. (**B**) Our workflow from data generation, to rapid testing and communication, following principles of Open Science. (**C**) A maximum-likelihood phylogenetic tree showing 1048 SARS-CoV-2 genomes, including the first 200 SARS-CoVi2 genomes sequenced by our group. (**D**) The phylogeographic reconstruction of the genomes shown in (C) reveals the potential sources of introductions and patterns of viral spread within Connecticut. The size of the nodes represents the number of genomes sampled in the corresponding area, and the edges depict viral spread events between these areas. The results, which are embedded into our reports (available on covidtrackerct.com/portfolio/current/), can also be viewed standalone by loading output files hosted on GitHub (https://github.com/grubaughlab/CT-SARS-CoV-2).

We launched the Yale SARS-CoV-2 Surveillance Initiative to uncover the patterns of the spread and ongoing transmission of SARS-CoV-2 in Connecticut, USA. The public face of our initiative is CovidTrackerCT (covidtrackerct.com) (Fig 1A), a platform launched in late April 2020 to rapidly communicate our findings and translate our research into tangible public health benefits. CovidTrackerCT is based on the aforementioned framework (Box 1; Fig 1B). We present easily digestible descriptions of our analysis and major conclusions in both English and Spanish, alongside interactive figures facilitated by the Nextstrain visualization toolkit (Fig 1C and 1D). From April-July, 2020, our platform has attracted nearly 6,000 visitors.

Speed is emphasized, and results are made public within days of generating complete genomes (Fig 1C and 1D). To achieve a balance with accuracy, any caveats and limitations are disclosed. Also, supplemental information explaining genomic epidemiology concepts and protocols used to generate the results are made available in specific subpages. Results presented are framed in an actionable way; for example, discussing how the patterns of viral spread from neighboring states and between local towns highlight the importance of coordinated testing, contact tracing, and other non-pharmaceutical interventions.

The primary audience of the platform are those for whom the results are most consequential: state residents and those shaping public policy at the state and local level. The format and content of the weekly reports can be adapted in response to new information, needs, and tools. It is important to note that such science communication platforms are not intended to replace more traditional pathways of sharing this work, for example, peer-reviewed research papers, but serve a separate purpose of sharing it in real time, in a jargon-free way, to take advantage of the speed at which we can now generate and analyze data.

## Conclusions

Many US states, including Connecticut, are looking to establish policies to mitigate virus spread for the coming months and years. As the outward-facing arm of the Yale SARS-CoV-2 Genomic Surveillance Initiatives, CovidTrackerCT played a role in informing local, state, and (Northeastern US) regional policy. Specifically, those behind the initiative have been contacted by mayors, state senators, and governors about our conclusions. After demonstrating how SARS-CoV-2 spread in Connecticut is highly influenced by outbreaks in surrounding areas, especially New York City, our results likely influenced policymakers to (***1***) enact greater coordination of control efforts between the adjacent states of Connecticut, New York, and New Jersey and (***2***) implement quarantine procedures for travelers arriving from affected states. Approaches to establishing these policies and communicating the reasoning behind them must be driven by data, including genomic data. Clear communication directly from experts provides an opportunity for closer interaction and trust-building between researchers and the public and helps protect against the spread of misinformation.

## Ethics statement

Collection of clinical specimens at the Yale-New Haven Hospital for sequencing was approved by the Institutional Review Board of the Yale Human Research Protection Program (FWA00002571, Protocol ID. 2000027690).
